# miR-155 Inhibits Nucleus Pulposus Cells' Degeneration through Targeting ERK 1/2

**DOI:** 10.1155/2016/6984270

**Published:** 2016-08-18

**Authors:** Dongping Ye, Libing Dai, Yicun Yao, Shengnan Qin, Han Xie, Wen Wang, Weiguo Liang

**Affiliations:** Guangzhou City Red Cross Hospital, The Fourth Affiliated Hospital of the Medical College, Jinan University, Guangzhou 510220, China

## Abstract

We first investigated the difference in microRNA expression between normal NP cells and degenerative NP cells using gene chip. We have found that the expression of ERK1/2 was decreased with overexpression of miR-155 in normal nucleus pulposus cell. Expression of ERK1/2 was increased with inhibition of miR-155. Overexpression or inhibition of miR-155 had no effects on the expression level of mRNA ERK1/2 in nucleus pulposus cell, which showed that miR-155 affected the expression of pERK1/2 after transcription of ERK1/2 mRNA indicating that ERK1/2 was a new target protein regulated by miR-155. In the degeneration of intervertebral disc, inhibited miR-155 decreased the expressions of extracellular main matrix collagen II and glycosaminoglycan and increased expression of ERK1/2. Taken together, our data suggested that miR-155 was the identified miRNA which regulated NP cells degenerated through directly targeting ERK1/2.

## 1. Background

The intervertebral disc (IVD) which plays an important role in supporting loading of the spine is a fibrocartilage tissue that lies between vertebrae [1]. Degeneration of the IVD with ageing is commonly associated with low back pain [1]. The IVD is composed of the outer lamellar annulus fibrous (AF) and the central gelatinous nucleus pulposus (NP) [2]. The nucleus pulposus cells produce proteoglycan and type II collagen. The degradation of proteoglycan and type II collagen in extracellular matrix (ECM) content under pathological conditions is frequently observed in NP tissues [2]. Understanding the pathogenesis of IVD degeneration will help in inhibiting the degeneration of the IVD.

microRNAs (miRNAs) can bind to the 3′-untranslated region (3′UTR) of their target mRNA to repress gene expression at the posttranscriptional level [3, 4]. miRNAs regulate about 30% of human protein-coding genes expression [3]. Multiple miRNAs have been identified to regulate the complex genes expression in various human* in vitro *cells [3]. However, the effects of miRNAs expression in nucleus pulposus cells' degeneration have not been well characterized and are, therefore, particularly interesting to be elucidated [3].

Gene chip technology was applied to detect different expressions of miRNAs between human normal and degenerative nucleus pulposus cells. There were a range of 29 miRNAs which had been found by Wang et al. miR-155 was chosen for the study and they had found that the target genes of miR-155 were FADD and Caspase-3. FADD and Caspase-3 are the key proteins in the apoptosis pathway mediated by Fas/FasL. Downregulation of miR-155 in nucleus pulposus cells can upregulate the expression of FADD and Caspase-3 which are induced to increase the apoptosis of nucleus pulposus cells. Contradictory result was obtained by upregulating miR-155 [4].

We have also found that miR-155 was downregulated during NP cells degeneration. The expression of microRNA-155, which would decrease in degenerative nucleus pulposus cell, has been verified by using gene chip and RT-PCR in this dissertation. The predicted target genes of miR-155 by TargetScan software include MAPK signal pathway, whereas Kras-Raf1-MEK1/2-ERK1/2 signal pathway was fully characterized in NP cells.

Therefore, we used mimic-155 and inhibitor-155 to overexpress and decrease the miR-155 expression, using RT-PCR and western blot to detect Kras, RAF1, MEK1/2, and ERK1/2 genes and proteins expression. The study investigates the relationship between miR-155 and MAPK pathways. Moreover, this study provides a novel potential therapeutic target for intervertebral disc degeneration.

## 2. Methods

### 2.1. Isolation and Passage of Normal and Degenerated NP Cells

Normal NP cells were obtained from three scoliosis patients who underwent orthopaedic surgery (two cases aged 13 years, one case aged 15 years); degenerative NP cells were obtained from three lumbar disc herniation patients who underwent orthopaedic surgery (two cases aged 65 years, one case aged 70 years). All samples were assessed histologically and graded for the degree of degeneration according to the method by Sive et al. [5]. Samples of the three normal discs were defined as histological grade 1, whereas samples of the three degenerated discs were defined as histological grades 8, 9, and 10. The NP tissues were removed and soaked in saline containing penicillin-streptomycin antibiotics for 10 min; the NP tissues were gently separated from the disc with a curette and then washed three or four times with saline until no blood was visible. Tissues were then cut into 1 × 1 × 1 mm pieces with ophthalmic scissors, placed in a 100 mL beaker filled with 10 mL 2% collagenase II, and stirred for 60 min. The completely digested tissues were centrifuged at 1000 rpm for 10 min. The supernatant was aspirated, and the cells were dispersed with 1 mL DMEM containing 10% fetal calf serum. The cells were then cultured in a T25 tissue culture flask with 6 mL DMEM containing 10% fetal bovine serum at 37°C, saturated humidity, and 5% CO_2_ for 3 days.

### 2.2. miRNA Extraction

The total RNA from the collected cells was extracted using TRIzol reagent (Invitrogen, USA) according to the manufacturer's instructions. The total RNA was collected for real-time PCR (qRT-PCR) analysis.

Reverse transcription was carried out using a TaqMan microRNA reverse transcription kit (Applied Biosystems, Foster City, CA, USA) according to the manufacturer's protocol. Hsa-miR-155 was detected using TaqMan microRNA assays (Assay ID 000479, Applied Biosystems). Real-time quantification of cDNA was carried out on a 7900HT Fast Real-Time PCR System (Applied Biosystems).

Expression data were normalized according to the expression of the RNU48 reference DNA (Assay ID 001006, Applied Biosystems).

### 2.3. Mark of Sample RNA

The experimental samples of total RNA (including miRNA) were marked with FlashTag™ Biotin HSR RNA Labeling Kit (P/N 901911, Affymetrix), corresponding kit of Affymetrix miRNA chip, and standard operation procedure.

### 2.4. Chip Hybridization

Following standard operation procedure provided by Affymetrix miRNA chip and with the corresponding kit, GeneChip® Hybridization, Wash, and Stain Kit (P/N 900720, Affymetrix, Santa Clara, CA, US) and GeneChip Eukaryotic Hybridization Control Kit (P/N 900454, Affymetrix, Santa Clara, CA, US), the chip was hybridized in scrolling hybridization oven in 48°C in the next 16 hours, (Hybridization Oven 645, P/N 00-0331 (220 V), Affymetrix, Santa Clara, CA, US). After hybridization, the chip was washed in Fluidics Station 450 (P/N 00-0079, Affymetrix, Santa Clara, CA, US), the washing workstation, following standard operation procedure provided by Affymetrix.

### 2.5. Chip Scanning and Target Prediction

Chip result was scanned by using GeneChip Scanner 7G (Affymetrix, Santa Clara, CA, US). Command Console Software 3.2 (Affymetrix, Santa Clara, CA, US) was used to load the raw data. Qualified data through quality testing were normalized with Expression Console (Affymetrix, Santa Clara, CA, US), using RMA+DABG as algorithm.

Databases such as TargetScan, miRDB, miRanda, miRBase, and miRWalk database were applied to target prediction. The relationship between microRNA and ERK family would be studied selectively according to signal intensity and fold change of microRNA gene chip and depending on its target gene containing ERK family.

### 2.6. RNA Isolation and Real-Time PCR

Total RNA from cells was extracted with TRIzol reagent (Invitrogen) according to the manufacturer's instructions. First-strand cDNA was synthesized from 1 *μ*g of total RNA by incubating for 1 h at 42°C with Superscript III reverse transcriptase (Invitrogen, Mulgrave, Australia) following oligo (dT) priming. After reverse transcription reaction, qRT-PCR was performed by LightCycler 480 system (Roche, Mannheim, Germany) using SYBR Premix Ex TaqTM (Takara, Dalian, China) according to the manufacturer's instructions. All amplification processes were normalized by GAPDH. Data were analyzed using the comparison Ct (2^−ΔCt^) method to get the lg2 (microarray normalized signal). Each sample was analyzed in triplicate. The primer sequences used in this study are as follows:

*miR-155*

 GSP: 5′-GGGGTTAATGCTAATCGTGA-3′.
 Reverse sequence: 5′-CGCTTCACGAATTTGCGTGTCAT-3′.
 U6 Forward sequence: 5′-CTCGCTTCGGCAGCACA-3′.
 Reverse sequence: 5′-CGAATTCTAGAGCTCGAGGCAGG-3′.

GSP is the specific primer of the corresponding microRNA.

The primer sequences for type II collagen are as follows:

 Forward: 5′-TGGTGGCTTCCATTTCAGCT-3′.
 Reverse: 5′-TGTTCTGGGAGCCTTCCGT-3′.

The primer sequences for aggrecan are as follows:

 Forward: 5′-AGCCTGCGCTCCAATGACT-3′.
 Reverse: 5′-GGAACACGATGCCTTTCACC-3′.

### 2.7. Western Blotting

Western blotting was used to confirm the expression of proteins. Proteins were separated in 10% sodium dodecyl sulphate-polyacrylamide gel electrophoresis gel by electrophoresis (90 V, constant voltage) for 120 min and then by transferring to a 0.45-mM PVDF membrane. The transferred membranes were incubated with anti-ERK1/2 (1 : 500 dilution), anti-pERK1/2 (1 : 500 dilution), anti-Kras (1 : 500 dilution), anti-Raf1 (1 : 500 dilution), anti-pRaf1 (1 : 500 dilution), anti-MEK1/2 (1 : 500 dilution), anti-pMEK1/2 (1 : 500 dilution), and anti-mouse-HRP (1 : 3000 dilution) antibodies. All antibodies were purchased from Abcam (Cambridge, MA, USA). The western blot signals were detected by ECL reagents and imaged for analysis.

### 2.8. Detection of Collagen II and Aggrecan Content in Extracellular Fluid by ELISA Assay

The expression of type II collagen and aggrecan in extracellular fluid was determined by ELISA assays using an ELISA kit purchased from USCNLIFE (Wuhan, China). The sample groups consisted of blank wells, standard wells, and detected sample wells.

### 2.9. Transfecting miR-155 Mimics and Inhibitor Using Liposomes Method

 hsa-miR-155hsa-miR-155
 mimics: 5′-UUAAUGCUAAUCGUGAUAGGGGU-3′, 5′-CCCUAUCACGAUUAGCAUUAAUU-3′
 hsa-miR-155 inhibitor: 5′-ACCCCUAUCACGAUUAGCAUUAA-3′

The research was divided into three groups: transfecting hsa-miR-155 mimics (group A), transfecting hsa-miR-155 inhibitor (group B), and transfecting blank liposomes only (group C). Normal nucleus pulposus cells were cultured under the condition of 37°C and 5% CO_2_ in culture medium containing 10% fetal calf serum. The day before transfection, well-grown cells were trypsinized, then harvested, and counted, spreading into the pore plate equably. 6 × 104 cells were inoculated into every pore. In the next day, cells were transfected when they were totally adherent and the density reached 70%. The procedure is as follows. Cell inoculation: the day before transfection, cells were inoculated into 96 pore plates (3000/pore) and were cultured in a complete culture medium without antibody and were transfected when the cell density reached 40~60%. Rapid centrifugation of the ordered miRNA control, miR-155 mimics, miR-155 inhibitors, and their contrasts was done, and then corresponding amount of RNase-free dd H_2_O was prepared. The transfection reagent Lipofectamine™ 2000 reverse was blended before being used. Then, 0.5 *μ*L transfection reagent was diluted with 25 *μ*L Opti-MEM® I Reduced Serum Medium culture medium and then maintained at room temperature for 5 min. Notice: the next step proceeded in 25 min. 0.375 *μ*L oligonucleotides (final concentration 50 nM) were put into 25 *μ*L Opti-MEM I Reduced Serum Medium culture medium and then blended gently. The diluted oligonucleotides were mixed with Lipofectamine 2000 and then cultured at room temperature for 20 min. 50 *μ*L mixture of plasmid oligonucleotide and transfection reagents was placed into culture hole containing cells and culture medium. Cells were cultured under condition of 37°C and 5% CO_2_ for 6 hours.

### 2.10. Statistical Analyses

All numerical data are expressed as the mean ± SD (statistical differences) among groups that were analyzed by one-way analysis of variance with a* post hoc* test to determine group differences in the study parameters. All statistical analyses were performed with SPSS software, version 13.0. Statistical differences between two groups were determined by Student's* t*-test. *P* < 0.05 was considered statistically significant. All the experiments were repeated 3 times.

## 3. Results

Compared to normal nucleus pulposus cells, 86 microRNAs were upregulated and 77 microRNAs were downregulated in degenerative nucleus pulposus cells. Applied critical review of literature and bioinformatics analysis showed that microRNA was chosen for research with the signal at both sides being greater than 8 and the multiple between them being greater than 2, and their predicted target gene was composed of ERK family members. MiR-155 was chosen for further studies. MiR-155 was highly expressed in normal nucleus pulposus cells and was commonly expressed in degenerative nucleus pulposus cells.

**Figure 1:**
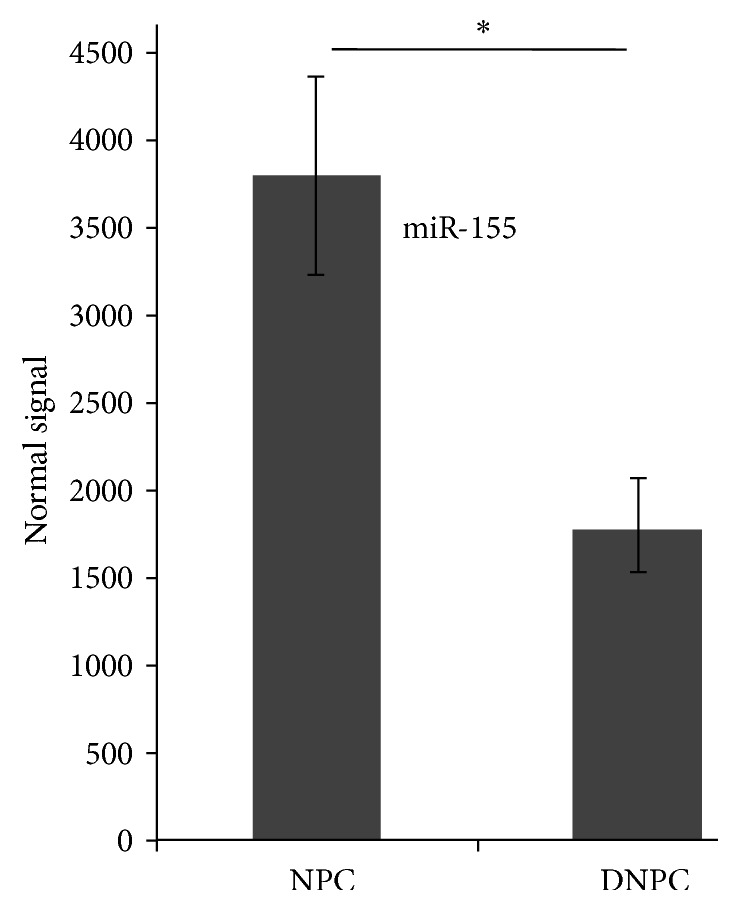
RT-PCR analysis of miR-155. miR-155 was highly expressed in normal nucleus pulposus cells and was lowly expressed in degenerative nucleus pulposus cells. Normal signal: data were analyzed using the comparison Ct (2^−ΔCt^) method to get the lg2 (microarray normalized signal). ^*∗*^ *p* < 0.05.

Using bioinformatic analysis, we have found that miR-155 was downregulated during NP cells' degeneration. The expression of miR-155, which decreased in degenerative nucleus pulposus cell, has been verified by using RT-PCR in this dissertation (Figure 1).

**Figure 2:**
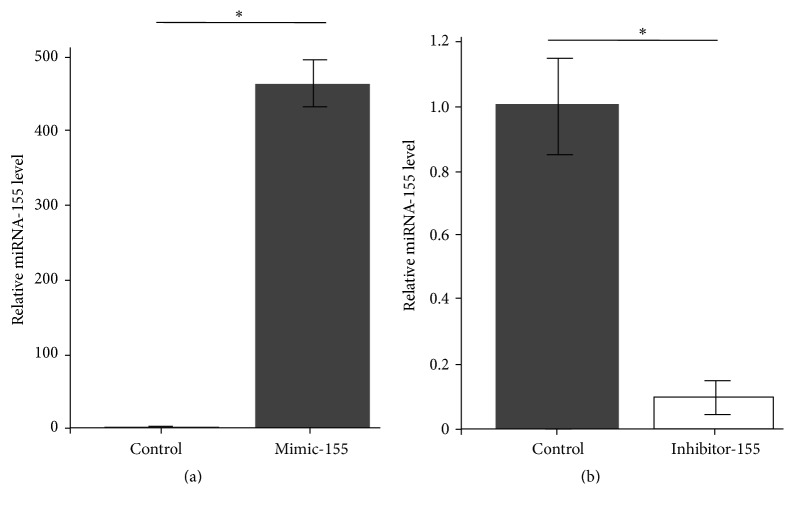
MiR-155 was overexpressed and decreased with hsa-miR-155 mimics and hsa-miR-155 inhibitor in normal nucleus pulposus cell (*n* = 3). ^*∗*^ *p* < 0.05.

Overexpression and inhibition of miR-155 were observed with hsa-miR-155 mimics and inhibitor; miR155 was overexpressed by hsa-miR-155 mimics about 450 times whereas it is inhibited by hsa-miR-155 inhibitor about 9 times. The results suggested that it was successful to overexpress and inhibit miR-155 (Figures 2(a) and 2(b)).

**Figure 3:**
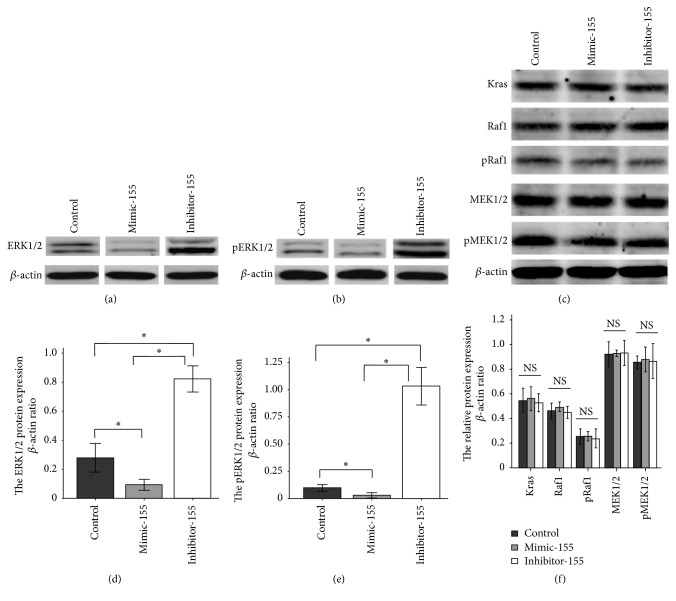
Western blot analysis of Raf1-MEK1/2-ERK1/2 pathway in relation to miR-155. Expression of ERK1/2. pERK1/2 decreases with overexpression of microRNA-155. When miR-155 is inhibited, expression of ERK1/2 and pERK1/2 increases (*n* = 3). ^*∗*^ *p* < 0.05.

Expression of ERK1/2 and pERK1/2 decreased with overexpression of miR-155 in normal nucleus pulposus cell (Figures 3(a), 3(b), 3(d), and 3(e)). Expression of ERK1/2 and pERK1/2 increased with inhibition of miR-155 (Figures 3(a), 3(b), 3(d), and 3(e)). There was no effect on the Kras, Raf1, p-Raf, MEK1/2, and pMEK1/2 proteins expression when overexpressing and inhibiting the miR-155 (Figures 3(c) and 3(f)).

**Figure 4:**
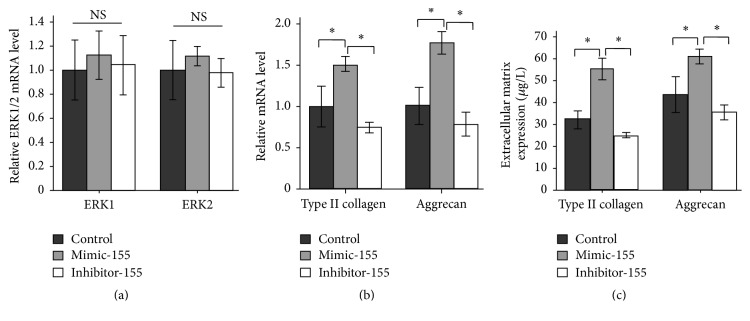
RT-PCR analysis of ERK1/2, type II collagen, and aggrecan mRNA expression in relation to miR-155. Overexpression or inhibition of miR-155 has no effects on expression level of mRNA ERK1/2 in nucleus pulposus cell. Inhibited miR-155 decreases the expressions of extracellular main matrix collagen II and glycosaminoglycan while increasing expression of ERK1/2 (*n* = 3). ^*∗*^ *p* < 0.05.

Overexpression or inhibition of miR-155 had no effects on the expression level of mRNA ERK1/2 in nucleus pulposus cell (Figure 4(a)), which showed that miR-155 affected the expression of pERK1/2 after transcription of ERK1/2 mRNA indicating that ERK1/2 was a new target protein regulated by miR-155.

It has been declared that nucleus pulposus cells would degenerate by activating ERK1/2 pathway [2]. Inhibited miR-155 decreased the expressions of extracellular main matrix collagen II and glycosaminoglycan and increased expression of ERK1/2 (Figures 4(b) and 4(c)). It was thought that miR-155 could affect the nucleus pulposus cells degeneration through target gene ERK1/2.

## 4. Discussion

MiR-155 is a multifunctional miRNA which plays an important role in regulating immune function in inflammation [6]. The miR-155 located at chromosome 21q21.3 was found from B-Cell Integration Cluster (BIC) [7]. The expression of miR-155 increased in activated B cells, T cells, and monocyte/macrophage [8]. In addition, the study had found that miR-155 exists in synovioblast and mononuclear cells in rheumatoid arthritis [9]. Therefore, miR-155 plays an important role in inducing inflammatory reaction and immune response.

It has been found that potential molecular mechanism increased apoptosis and expression profile of miRNAs in the degeneration of the human intervertebral disc [3]. Meanwhile, it was confirmed that Caspase-3 was the new target protein regulated by miR-155 [3]. The fact indicated that miR-155 plays an important role in the pathogenesis of degeneration of human intervertebral disc. We have also found that miR-155 was downregulated during NP cells degeneration. The expression of microRNA-155, which would decrease in degenerative nucleus pulposus cell, has been verified by using gene chip and RT-PCR in this dissertation. It is consistent with the previous study. The predicted target genes of miR-155 by TargetScan software include MAPK signal pathway, whereas Kras-Raf1-MEK1/2-ERK1/2 signal pathway was fully characterized in NP cells. So we have focused on the Kras-Raf1-MEK1/2-ERK1/2 signal pathway since it had been declared that nucleus pulposus cells would degenerate by activating this signal pathway.

We have found that the expression of ERK1/2 and pERK1/2 decreased with overexpression of miR-155 in normal nucleus pulposus cell. The expression of ERK1/2 and pERK1/2 increased with inhibition of miR-155. There was no effect on the Kras, Raf1, p-Raf, MEK1/2, and pMEK1/2 proteins expression while overexpressed or inhibited the miR-155. Overexpression or inhibition of miR-155 had no effects on the expression level of mRNA ERK1/2 in nucleus pulposus cell, which showed that miR-155 affected the expression of pERK1/2 after transcription of ERK1/2 mRNA indicating that ERK1/2 was a new target protein regulated by miR-155.

ERK1/2 is a protein kinase separated and identified by Spoerl et al. in the early 1990s [10]. Their relative molecular weights are 44 kD and 42 kD, respectively, and they share a high homology of 90% [10, 11]. ERK1/2 mediated serine/threonine kinase of proline, which is able to make serine/threonine next to proline phosphorylated [12]. The biological effect of the ERK1/2 was through multistage kinase cascade of MAPK signal transduction pathway [12, 13].

Risbud et al. found that ERK1/2 and P38 signals were continuously phosphorylated in mice nucleus pulposus cells in hypoxic environment [14]. Further research demonstrated that the activation of ERK1/2 of nucleus pulposus cells in high osmotic environment could activate its downstream transcription factor ELK-1 [14]. In the progress of degeneration of nucleus pulposus cells induced by tumor necrosis factor-*α* (TNF-*α*), TNF-*α* could influence the progress of the disease and the formation of new vessels by upregulating metalloproteases, such as matrix metalloproteinases 2 [15, 16].

We have found that overexpression or inhibition of miR-155 has no effects on the expression level of mRNA ERK1/2 in nucleus pulposus cell. Inhibited miR-155 decreases the expressions of extracellular main matrix collagen II and glycosaminoglycan while increasing expression of ERK1/2. According to the mentioned above, we speculated that inhibited miR-155 decreased the expressions of extracellular main matrix collagen II and glycosaminoglycan and increased expression of ERK1/2. It was thought that miR-155 could affect the nucleus pulposus cells degeneration through target gene ERK1/2.

## 5. Conclusion

Taken together, our data suggested that miR-155 was the identified miRNA that regulated NP cells degenerated through directly targeting ERK1/2, and therapeutic overexpression of miR-155 may be an efficient anabolic strategy for intervertebral disc degeneration.

## Additional Points

*Availability of Data and Materials.* All relevant raw data will be freely available to any scientist wishing to use them for noncommercial purposes, without breaching participant confidentiality.
